# Role of the Dopaminergic System in the Striatum and Its Association With Functional Recovery or Rehabilitation After Brain Injury

**DOI:** 10.3389/fnins.2021.693404

**Published:** 2021-06-24

**Authors:** Antonio Verduzco-Mendoza, Paul Carrillo-Mora, Alberto Avila-Luna, Arturo Gálvez-Rosas, Adriana Olmos-Hernández, Daniel Mota-Rojas, Antonio Bueno-Nava

**Affiliations:** ^1^Ph.D. Program in Biological and Health Sciences, Universidad Autónoma Metropolitana, Mexico City, Mexico; ^2^Division of Biotechnology-Bioterio and Experimental Surgery, Instituto Nacional de Rehabilitación-Luis Guillermo Ibarra Ibarra, Mexico City, Mexico; ^3^Division of Neurosciences, Instituto Nacional de Rehabilitación-Luis Guillermo Ibarra Ibarra, Mexico City, Mexico; ^4^Neurophysiology, Behavior and Animal Welfare Assessment, DPAA, Universidad Autónoma Metropolitana, Mexico City, Mexico

**Keywords:** dopamine, corticostriatal pathway, functional recovery, traumatic brain injury, dopamine receptors

## Abstract

Disabilities are estimated to occur in approximately 2% of survivors of traumatic brain injury (TBI) worldwide, and disability may persist even decades after brain injury. Facilitation or modulation of functional recovery is an important goal of rehabilitation in all patients who survive severe TBI. However, this recovery tends to vary among patients because it is affected by the biological and physical characteristics of the patients; the types, doses, and application regimens of the drugs used; and clinical indications. In clinical practice, diverse dopaminergic drugs with various dosing and application procedures are used for TBI. Previous studies have shown that dopamine (DA) neurotransmission is disrupted following moderate to severe TBI and have reported beneficial effects of drugs that affect the dopaminergic system. However, the mechanisms of action of dopaminergic drugs have not been completely clarified, partly because dopaminergic receptor activation can lead to restoration of the pathway of the corticobasal ganglia after injury in brain structures with high densities of these receptors. This review aims to provide an overview of the functionality of the dopaminergic system in the striatum and its roles in functional recovery or rehabilitation after TBI.

## Introduction

Traumatic brain injury (TBI) is a main cause of disability; approximately 2% of people worldwide suffer from a TBI-related disability (Bales et al., 2009). Currently, TBI is defined as “an alteration in brain function, or other evidence of brain pathology, caused by an external force” (Menon et al., 2010). Anatomical damage, neurological deficits and mental status are common indices used to categorize TBI as mild, moderate or severe (Corrigan et al., 2010). One of the structures that is typically injured in TBI is the cerebral cortex, which is anatomically connected with other brain regions, such as the striatum, thalamus, pons and cerebellum, by afferent and efferent axons in pathways such as the corticobasal ganglia-thalamocortical and cerebellothalamocortical pathways (Daskalakis et al., 2004; Bostan and Strick, 2010; Mendoza and Merchant, 2014).

The striatum is the main input nucleus of the basal ganglia and receives glutamatergic afferents, such as motor, oculomotor, executive/associative and emotion-/motivation-related afferents, from diverse cortical areas (Reiner et al., 2010; Mathai and Smith, 2011; Kandel et al., 2013). Corticostriatal glutamatergic inputs are bilateral but have an ipsilateral predominance in the striatum (Reep et al., 2003; Lei et al., 2004; Wu et al., 2009; Reiner et al., 2010) and are critically modulated by dopamine (DA) produced by the substantia nigra *pars compacta* (SNc) (Bjorklund and Dunnett, 2007). TBI is associated with effects on dopaminergic transmission at the level of the striatum (Bales et al., 2009; Karelina et al., 2017; Jenkins et al., 2018; Plummer et al., 2018), such as reductions in the synthesis and release of striatal DA (Wagner et al., 2005b; Shin et al., 2011).

The literature supports the beneficial effects of dopaminergic drugs in patients with TBI, confirming that they accelerate functional recovery and rehabilitation after brain injury (Harmon and Boyeson, 1997; Bales et al., 2009; Frenette et al., 2011, 2012; Osier and Dixon, 2016a; Carrillo-Mora et al., 2017; Munakomi et al., 2017; Duong et al., 2018; Plummer et al., 2018; Loggini et al., 2020). The mechanisms of action of dopaminergic drugs administered to patients with TBI include blockade of the DA transporter (DAT), inhibition of DA reuptake and facilitation of DA synthesis, and the drugs most highly recommended by the Neurotrauma Foundation are methylphenidate (MPD), amantadine, and bromocriptine (Bales et al., 2009). However, administration of these drugs to patients with TBI is recommended only for symptomatic therapy to enhance attentional function, processing speed, and executive function (Bales et al., 2009).

The mechanisms of action of dopaminergic drugs have not been completely clarified, partly because dopaminergic receptor activation leads to restoration of neuronal circuits after injury to brain structures with high densities of these receptors. This review aims to provide an overview of the functionality of the dopaminergic system in the dorsal striatum and its role in functional recovery or rehabilitation after TBI.

## Organization of the Corticostriatal Pathway

The corticostriatal pathway originates mainly in the motor and premotor cortices of the brain from pyramidal neurons located in layers V and III (Mathai and Smith, 2011). Glutamatergic cortical afferents target dendritic spines of medium spiny neurons (MSNs) in the striatum (Bolam et al., 2000; Surmeier et al., 2007; Kreitzer and Malenka, 2008; Silberberg and Bolam, 2015). In addition, the striatum receives glutamatergic afferent inputs from the thalamus, subthalamic nucleus, pedunculopontine nucleus, hippocampus, and amygdala (Smith and Parent, 1986; Doig et al., 2010; Lodge and Grace, 2011; Mathai and Smith, 2011; Koshimizu et al., 2013; Haber, 2016; Wouterlood et al., 2018; Assous et al., 2019). The main glutamatergic inputs to the MSNs are derived from the brain cortex and thalamus (Haber, 2016). Cortical inputs to the striatum are dominant with respect to thalamic inputs in terms of axonal density and synaptic interactions with MSNs (Lei et al., 2004; Smith et al., 2004; Reiner et al., 2010; Silberberg and Bolam, 2015; Haber, 2016). These corticostriatal inputs are bilateral but have an ipsilateral predominance (Reep et al., 2003; Lei et al., 2004; Wu et al., 2009; Reiner et al., 2010).

In rodents, the striatum receives corticostriatal inputs from intratelencephalic (IT) neurons and pyramidal tract (PT) neurons (Mathai and Smith, 2011; Shepherd, 2013). IT afferents are principally derived from neurons located in layer III and in upper layer V of the brain cortex (Lei et al., 2004; Mathai and Smith, 2011). In the striatum, afferent inputs from IT and PT cortical neurons target both direct and indirect pathways (Doig et al., 2010; Mathai and Smith, 2011; Silberberg and Bolam, 2015; Haber, 2016). The striatal dorsolateral area receives cortical input from the sensory-motor region, the striatal central and dorsomedial areas receive inputs from the associative cortical region, and the striatal ventromedial area receives input from the limbic region (Haber, 2016; Kupferschmidt et al., 2017).

## Distribution and Function of DA Receptors

**FIGURE 1:**
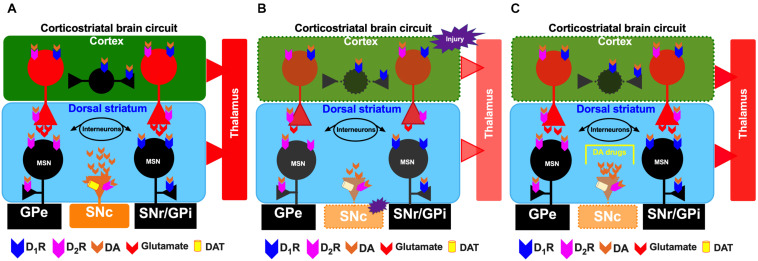
Schematic representation of the corticostriatal pathway under normal conditions **(A)**, under conditions of cortical injury **(B)**, and during the injury recovery process/the proposed effects of administration of dopaminergic drugs **(C)**. For the circles, triangles and lines, red indicates excitatory projections, whereas black indicates inhibitory projections. The color orange represents the substantia nigra pars compacta (SNc). The dotted lines indicate depletion by injury in the cerebral cortex and SNc. SNr, substantia nigra pars reticulata; GPi, internal segment of the globus pallidus; GPe, external segment of the globus pallidus; STN, subthalamic nucleus.

The DA receptor family includes D_1_-like (D_1_ and D_5_) and D_2_-like (D_2_, D_3_, and D_4_) subtypes (Bunzow et al., 1988; Sunahara et al., 1991; Van Tol et al., 1991; Missale et al., 1998; Vallone et al., 2000). In the cerebral cortex, autoradiographic localization studies have shown that both rodent and primate cortices contain D_1_ receptors (D_1_Rs) and D_2_ receptors (D_2_Rs) (Camps et al., 1990), but the density of D_1_Rs is 10–20-fold higher than that of D_2_Rs (Lidow et al., 1991). In addition, the highest ratio of mRNA expression of D_1_Rs compared with D_2_Rs is found in the prefrontal cortex (Santana and Artigas, 2017), where both D_1_Rs and D_2_Rs are expressed in glutamatergic and GABAergic neurons (Figure 1A; Santana and Artigas, 2017). As previously reported (Abekawa et al., 2000), D_1_R activation reduces extracellular glutamate and GABA levels in the medial prefrontal cortex. In one study, D_1_R activation of interneurons led to decreased activity of the corticostriatal pathway (Muly et al., 1998). Other studies have documented the localization of D_1_Rs in corticostriatal pyramidal neurons, while some authors reported that the effects of D_2_R activation are confined to type I corticopontine neurons of layer V (Gaspar et al., 1995; Leyrer-Jackson and Thomas, 2017). Similar to the case in the striatum, D_2_Rs in the prefrontal cortex are restricted to specific neural populations (Gee et al., 2012). In addition, D_2_Rs are localized in the axons of dopaminergic afferents (Bjorklund and Dunnett, 2007).

In the striatum, a subpopulation of MSNs known as striatonigral neurons express D_1_-like receptors, predominantly D_1_Rs, at high levels (Bergson et al., 1995; Le Moine and Bloch, 1995; Surmeier et al., 1996); however, D_2_Rs are selectively expressed in a second subpopulation, the striatopallidal MSNs (Le Moine and Bloch, 1995; Valjent et al., 2009). Activation of pre- and postsynaptic D_1_Rs and D_2_Rs is associated with modulation of corticostriatal glutamatergic transmission and synaptic plasticity in corticostriatal synapses (Kerkerian et al., 1987; Hsu et al., 1995; López De Maturana and Sánchez-Pernaute, 2010). Functionally, striatal D_1_Rs and D_2_Rs, but not extrastriatal DA receptors, are responsible for dopaminergic motor stimulation (Wang and Zhou, 2017). As shown in our recent studies, D_1_R activation maintains motor coordination and balance in both normal and injured rats (Avila-Luna et al., 2018a, b).

## Neuronal Death and Axonal Injury in Neural Groups After TBI

Primary injury is characterized locally by destruction of brain tissue, including neuronal and glial destruction originating from intracranial hemorrhage, epidural and subdural hematomas, brain contusions and direct mechanical injury (Eakin et al., 2013; Loane and Kumar, 2016; Lu et al., 2019; Shi et al., 2019). Primary brain injury leads to secondary events, including hypoxia, excitotoxicity, free radical generation, vascular dysfunction, apoptotic cell death, the inflammatory response and brain edema (Eakin et al., 2013; Chandran et al., 2018; Lu et al., 2019; Ng and Lee, 2019; Shi et al., 2019). As consequences of this damage, the concentrations of neurotransmitters, including monoamines (DA, norepinephrine, and serotonin) (Massucci et al., 2004; Bueno-Nava et al., 2008, 2010b; Huang et al., 2014a; Chen et al., 2015b, 2017b) and amino acids (GABA and glutamate) (Krobert et al., 1994; Bueno-Nava et al., 2008, 2010a; Shin et al., 2011; Guerriero et al., 2015), change in other brain structures. Glutamate release is associated with excitotoxicity following TBI, which leads to neuronal death (Guerriero et al., 2015), and elevated glutamate levels that persist over time (up to 4 days) are associated with a high mortality rate (23.6%) or with poor functional recovery in patients with injury (Chamoun et al., 2010).

Other consequences of brain injury are alterations in adjacent or distant neural groups with respect to the brain cortex, including structures that are anatomically related to the injured site, such as the striatum, cerebellum, pons, and hippocampus (Dunn-Meynell and Levin, 1997; Reep and Corwin, 1999; Van Vleet et al., 2003; Park et al., 2007; Bueno-Nava et al., 2008, 2010b; Doig et al., 2010; Kernie and Parent, 2010; Wiley et al., 2016; Shipp, 2017; Gálvez-Rosas et al., 2019). Therefore, brain injury results in disruption of the connectivity of the corticostriatal, corticohypothalamic, corticopontocerebellar, and cerebellothalamocortical pathways (Leergaard, 2003; Daskalakis et al., 2004; Shepherd, 2013). Other neural groups, such as the dopaminergic, noradrenergic, histaminergic and serotonergic systems, are altered by local axonal injury in brain contusions and direct mechanical injury (Bueno-Nava et al., 2008, 2010b; Eakin et al., 2013; Jenkins et al., 2016, 2018; Avila-Luna et al., 2019; Liao et al., 2019; Dougherty et al., 2020).

## Striatal Dopaminergic Disruption After TBI

Primary cortical injury also involves local destruction of dopaminergic axons, a critical factor in the disruption of striatal dopaminergic signaling after TBI (Chen et al., 2017a; Karelina et al., 2017; Jenkins et al., 2018; Lu et al., 2019; Dougherty et al., 2020). As mentioned above, several events occur during secondary injury, but one event of interest in our review is the disruption of the nigrostriatal dopaminergic system at the striatal level (Figure 1B).

In the dorsal striatum, TBI is associated with effects on dopaminergic transmission on the side ipsilateral to the injury, such as reductions in the synthesis and release of striatal DA (Figure 1B; Wagner et al., 2005b; Shin et al., 2011; Van Bregt et al., 2012; Huang et al., 2014a; Chen et al., 2015b), subacute and chronic deficits in tyrosine hydroxylase activity (Wagner et al., 2005b; Hutson et al., 2011; Shin et al., 2011) and decreases in DAT levels in the striatum ipsilateral and/or contralateral to the injury (Donnemiller et al., 2000; Wagner et al., 2005a, b, 2014; Karelina et al., 2017). All previously mentioned studies indicate injury to dopaminergic neuronal cell bodies in the SNc (Jenkins et al., 2018). Patients with moderate-severe TBI reduced striatal DAT levels in the caudate nucleus (Figure 1B; Jenkins et al., 2018).

**FIGURE 2:**
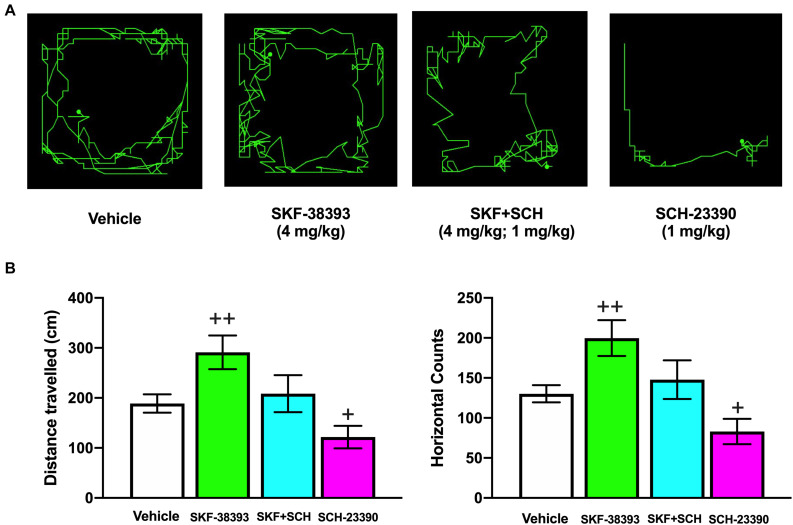
Effects of systemic administration of SKF-38393 alone, coadministration of SKF-38393 + SCH-23390 and administration of SCH-23390 alone on horizontal movement (distance traveled) and vertical movement (rearing behavior) in the spontaneous motor activity test. Representative maps for the distance traveled in each treatment group obtained at 20 or 40 min after drug administration **(A)**. Analysis of the groups **(B)**. The values are reported as the means ± SEMs. The statistical analyses were performed using one-way ANOVA followed by Dunnett’s test. **^+^***P* < 0.05 and **^++^***P* < 0.02 compared with the vehicle group. The results shown have been reported previously (Avila-Luna et al., 2018b).

In striatonigral MSNs, D_1_R activation increases the phosphorylation of the cAMP-regulated protein of 32 kDa (DARPP-32) at Thr34 (p-DARPP-32-Thr34) (Langley et al., 1997; Greengard et al., 1999; Calabresi et al., 2000; Undieh, 2010; Rangel-Barajas et al., 2015). Bales et al. (2011) showed that TBI decreases p-DARPP-32-Thr34 levels on both sides of the striatum and that the decrease in Thr34 phosphorylation is due to increased activity of protein phosphatase-1 (PP-1). DARPP-32 is a phosphoprotein regulated by DA, and one explanation for the decrease in DARPP-32 phosphorylation at Thr34 is a decrease in DA signaling, while the increased activity of PP-1 is associated with an alteration in PKA (Bales et al., 2011). Molecular studies in our laboratory on rats that had recovered from motor deficits at 192 h after cortical injury showed that D_1_R mRNA expression was decreased in the striatum ipsilateral to the injury site. This reduction in D_1_R mRNA expression was reversed by systemic administration of the D_1_R agonist SKF-38393 (2 mg/kg), an effect that was blocked by the D_1_R antagonist SCH-23390 (Gálvez-Rosas et al., 2019). In another study, we evaluated the effect of SKF-38393 on spontaneous motor activity in normal rats and found that it increased both the distance traveled and horizontal counts (Figure 2; Avila-Luna et al., 2018b). This increased locomotion was prevented by coadministration of SCH-23390; however, administration of SCH-23390 alone decreased both the distance traveled and the horizontal counts (Figure 2; Avila-Luna et al., 2018b). In this same study, we showed that D_1_Rs mediated the SCH-23390-induced deficits in motor coordination and spontaneous motor activity and that the effect was reversed upon subsequent administration of the full D_1_R agonist SKF-82958 (Avila-Luna et al., 2018a, b). However, the use of a D_1_R agonist did not accelerate motor recovery, although an intact striatum may be necessary to achieve recovery at 192 h postinjury (Avila-Luna et al., 2018a). Few studies have investigated neuronal injury in the striatum in humans and animals after TBI (Primus et al., 1997; Huang et al., 2018); more information on this type of injury is needed to better interpret the changes in D_1_R expression and function that occur in the striatum after TBI.

Another subpopulation of striatopallidal MSNs expresses D_2_-like receptors (D_2_Rs), which were recently reported to be altered in humans after TBI (Jolly et al., 2019) and have been studied and verified in animal models (Karelina et al., 2017). Immunohistochemistry of mouse tissues has indicated that D_2_R expression is decreased in the striatum ipsilateral to the injury site (Karelina et al., 2017), but such a decrease can be reversed by exercise on a motorized treadmill in rats that are exercised after injury, as reported by Ko et al. (2019). Two binding studies using single-photon or positron emission tomography (PET) have shown that striatal D_2_R binding is reduced in human patients with TBI (Donnemiller et al., 2000; Jolly et al., 2019). Another study has shown that caudate and putamen binding is reduced in patients with TBI and a length polymorphism near the D_2_R gene (Wagner et al., 2014). Low binding may be associated with nigrostriatal fiber loss (Donnemiller et al., 2000; Wagner et al., 2014).

The abovementioned evidence suggests that the dopaminergic system in the striatum is altered after TBI and that functional recovery is modulated by a D_1_R-dependent mechanism (Davis et al., 2007). Nevertheless, further studies on the striatum are needed to determine D_2_R function after TBI.

## Mechanisms of Action of Dopaminergic Drugs and Their Beneficial Effects on Brain Injury

Due to the high levels of D_1_R and D_2_R expression in the striatum and the strong connectivity of cortical glutamatergic afferents with both MSNs and dopaminergic afferents, alterations in this neuronal population may contribute to selective neuronal dysfunction and degeneration after brain injury (Jackson and Westlind-Danielsson, 1994; Le Moine and Bloch, 1995; Surmeier et al., 1996, 2007; Gerfen and Surmeier, 2011; Guerriero et al., 2015). Previous studies have shown that drugs that modulate dopaminergic transmission elicit favorable results during rehabilitation of patients with TBI, such as enhanced cognitive recovery, executive function, attentional function, processing speed and memory (Figure 1C; Harmon and Boyeson, 1997; Bales et al., 2009; Frenette et al., 2011, 2012; Osier and Dixon, 2016a; Plummer et al., 2018). Dopaminergic drugs administered to patients with TBI have various mechanisms of action, such as blockade of DAT, inhibition of DA reuptake and facilitation of DA synthesis. MPD, amantadine and bromocriptine are among the drugs most highly recommended by the Neurotrauma Foundation (Bales et al., 2009).

### Effects on DA Reuptake and the DAT

Catecholamine levels in the brain are increased in the first few hours after brain injury but decrease thereafter (Huger and Patrick, 1979; Dunn-Meynell et al., 1994; Levin et al., 1995; Osier and Dixon, 2016a). Striatal DA levels are altered in this way (Chen et al., 2015b, 2017b), and the alterations are accompanied by decreases in local and striatal DAT expression from the first to fourth weeks after cortical injury (Wagner et al., 2005a, b). The dorsal striatum is innervated by dopaminergic afferents from the SNc, which form synaptic contacts with dendritic spine necks on MSNs (Freund et al., 1984), and DAT is localized in the cell membrane of dopaminergic axons and terminals (Hersch et al., 1997). Focal cortical injury leads to loss of MSN spines and to decreases in synaptic contacts (Kemp et al., 1971; Cheng et al., 1997; Neely et al., 2007). In this context, imaging results in humans (Donnemiller et al., 2000) have been consistent with western blot findings (Wagner et al., 2005a, b), indicating that striatal DAT levels are decreased after brain injury (Wagner et al., 2005b). However, DAT inhibitors including amphetamine (Feeney et al., 1982; Hovda et al., 1987; Rau et al., 2016; Duong et al., 2018), MPD (Kline et al., 1994, Kaelin et al., 1996, Kline et al., 2000; Arciniegas et al., 2002; Weber and Lütschg, 2002; Demarchi et al., 2005; Wagner et al., 2007; Wortzel and Arciniegas, 2012; Ekinci et al., 2017), and amantadine (Arciniegas et al., 2002; Demarchi et al., 2005; Wu and Garmel, 2005; Wortzel and Arciniegas, 2012; Bleimeister et al., 2019; Okigbo et al., 2019; Loggini et al., 2020) have been used in animals and humans to evaluate the neuroprotective and symptomatic effects associated with improved recovery after brain injury. MPD and amantadine are clinically relevant treatments for TBI during the acute and chronic phases, including during the rehabilitative period (Arciniegas et al., 2002; Demarchi et al., 2005; Warden et al., 2006; Bales et al., 2009). In patients with TBI, administration of these drugs ameliorates neurocognitive impairments in attention, memory and executive function (Arciniegas et al., 2002; Rau et al., 2016). One explanation for the clinical use of DAT inhibitors despite the abnormally low DAT levels, is associated with changes in catecholamines after TBI (Osier and Dixon, 2016a), particularly decreases in catecholamine levels (Levin et al., 1995; Chen et al., 2015a, 2017b; Osier and Dixon, 2016a).

### DA Synthesis and Release

Functional neuroimaging results have shown that the substantia nigra is altered after cortical injury in TBI patients (Jenkins et al., 2018; Lu et al., 2019) and that this alteration can promote progressive degeneration of nigrostriatal dopaminergic neurons (Figure 1B; Hutson et al., 2011; Van Bregt et al., 2012). Subsequently, it leads to alterations in dopaminergic signaling in the striatum, including reductions in the synthesis and release of striatal DA (Wagner et al., 2005b; Shin et al., 2011; Van Bregt et al., 2012; Huang et al., 2014a; Chen et al., 2015b) and subacute and chronic deficits in tyrosine hydroxylase activity (Wagner et al., 2005b; Hutson et al., 2011; Shin et al., 2011). Levodopa is a DA precursor drug that is not only used to treat Parkinson’s disease but also administered to patients with stroke, as it exerts beneficial effects on motor recovery and cognitive function (Liepert, 2008; Seniów et al., 2009). In patients with TBI, levodopa is used to treat cognitive impairment (Arciniegas et al., 2002) and altered-consciousness states (Krimchansky et al., 2004; Matsuda et al., 2005). Clinical studies administering levodopa alone have been scarce and have included a limited number of patients (Demarchi et al., 2005); however, in some of these studies, administration of levodopa has improved the cognitive function and behaviors of all patients (Lal et al., 1988). Other studies have reported some beneficial effects of levodopa in clinical cases (Demarchi et al., 2005). For example, levodopa can be combined with other drugs, such as pramipexole, ropinirole, amantadine and bromocriptine, to improve apraxia (Jang et al., 2018; Choi et al., 2020). To exert its effects, levodopa increases striatal DA levels, which can lead to changes in signaling at DA receptors, including both D_1_Rs and D_2_Rs (De La Fuente-Fernandez et al., 2004; Aubert et al., 2005; Guigoni et al., 2005).

D_2_Rs are localized on dopaminergic axons (Bjorklund and Dunnett, 2007), in striatopallidal MSNs, in cholinergic interneurons and on corticostriatal terminals in the striatum (Le Moine and Bloch, 1995; Kreitzer and Malenka, 2008; Valjent et al., 2009). Bromocriptine is an ergot alkaloid and D_2_R-selective agonist that is known to enhance cognitive function in the chronic recovery period after brain injury (Ozga et al., 2018). In the traditional mechanism, DA D_2_ autoreceptor activation by agonist drugs at striatal presynaptic sites inhibits DA release (Brannan et al., 1993; L’hirondel et al., 1998; Schmitz et al., 2002, 2003) and synthesis (Schmitz et al., 2003; Yoshida et al., 2006). However, postsynaptic D_2_R activation modulates the functions of GABAergic MSNs and interneurons (Surmeier et al., 2007; Keeler et al., 2014). Notably, low (2.5 mg/kg) and medium (5 mg/kg) doses of bromocriptine increase DA release, an effect associated with the action of bromocriptine as a partial antagonist (Brannan et al., 1993). Compared with healthy control patients, who exhibit improved working memory, patients with mild TBI have altered responsivity to DA after administration of 1.25 mg of bromocriptine at 1 month after brain injury (Mcallister et al., 2011). In one study, administration of doses of 1.25 or 2.5 mg of bromocriptine 2 times daily resulted in enhanced functional recovery in patients with TBI who were in a vegetative state; however, the authors recognized the small sample size analyzed as a limitation of the study (Passler and Riggs, 2001). As mentioned above, bromocriptine is a D_2_R agonist but may also act as a partial antagonist (Lieberman and Goldstein, 1985), and some studies have shown a protective effect of bromocriptine against oxidative stress (Kline et al., 2004; Bales et al., 2009; Osier and Dixon, 2016a).

### Dopaminergic Mechanism of Action in Functional Recovery After Brain Injury

The mechanisms of action of the dopaminergic drugs used to treat TBI have not been completely clarified, primarily because dopaminergic receptor activation may lead to restoration of neuronal circuits after injury in brain structures with high densities of these receptors. Dopaminergic abnormalities following TBI are clearly associated with functional disability in animals and humans (Bales et al., 2009; Osier and Dixon, 2016a, b; Jenkins et al., 2018; Traeger et al., 2020; Tsuda et al., 2020), and drugs with dopaminergic actions are beneficial for functional recovery after brain injury (Meythaler et al., 2002; Matsuda et al., 2005; Frenette et al., 2012; Huang et al., 2014b; Duong et al., 2018; Lan et al., 2019; Okigbo et al., 2019; Loggini et al., 2020). In the striatum, activation of D_1_Rs and D_2_Rs on MSNs is associated with increased and decreased excitability, respectively, in response to corticostriatal glutamatergic inputs (Wickens and Wilson, 1998; Surmeier et al., 2007; Gerfen and Surmeier, 2011). The classic model of basal ganglia function (Figure 1A) suggests that glutamatergic corticostriatal projections are critical for the activity of striatonigral and striatopallidal MSNs that form the direct and indirect pathways in the basal ganglia, respectively (Albin et al., 1989). Alterations in dopaminergic function in the striatum are associated with motor disability, such as that observed in individuals with Parkinson’s disease and levodopa-induced dyskinesia (Calabresi et al., 2014; Bastide et al., 2015; Fundament et al., 2016; Schonfeld et al., 2017; Avila-Luna et al., 2019). Therefore, some components of TBI pathophysiology likely have a basis in striatal dopaminergic dysfunction based on clinical and experimental evidence obtained from the basal ganglia, particularly the dorsal striatum (Figure 1B; Donnemiller et al., 2000; Costa et al., 2006; Yan et al., 2007; Bales et al., 2009, 2011; Van Bregt et al., 2012; Osier and Dixon, 2016a; Karelina et al., 2017; Treble-Barna et al., 2017; Jenkins et al., 2018; Jolly et al., 2019). Similar to Parkinson’s disease, TBI leads to a hypodopaminergic state in the long term (Wagner et al., 2014; Jenkins et al., 2016; Karelina et al., 2017; Fridman et al., 2019). Various drugs have been used to counteract the adverse effects of the hypodopaminergic state, including DA agonists such as bromocriptine and amantadine, dopaminergic drugs used for Parkinson’s disease; stimulants; and reuptake inhibitors (Figure 1C and Table 1; Osier and Dixon, 2016a; Fridman et al., 2019).

**TABLE 1:** Main clinical effects of dopaminergic drugs on traumatic brain injury.

Drug	Mechanism of action	Clinical effects on traumatic brain injury	Side effects and limitations	References
**Amantadine**	Increases dopamine release and inhibits dopamine reuptake. Weak non-competitive NMDA receptor antagonist.	Post-concussion syndrome: decreases headache; exerts mild effects on memory, dizziness and behavioral disturbances.	Nausea, dizziness, insomnia, renal toxicity and decreased threshold for seizures.	Reddy et al., 2013; Carabenciov et al., 2019; Ma and Zafonte, 2020
		Behavioral symptoms: aggressiveness, agitation, apathy and irritability.		Gramish et al., 2017; Hammond et al., 2017; Ter Mors et al., 2019
		Ameliorates cognitive disturbances.		Ghate et al., 2018; Loggini et al., 2020
		Stimulates alertness in patients with altered states of consciousness such as unresponsive wakefulness syndrome or a minimally conscious state.		Giacino et al., 2012; Hadgu et al., 2020
		Stimulates of neuroplasticity processes in the acute stage.		Spritzer et al., 2015
**Modafinil**	Increases the levels of dopamine, norepinephrine, histamine, serotonin, and orexins.	Stimulates alertness in patients with altered states of consciousness such as unresponsive wakefulness syndrome or a minimally conscious state.	Nervousness, headache, dizziness, insomnia, nausea, rhinitis.	Dhamapurkar et al., 2017; Borghol et al., 2018; Repantis et al., 2021
		Ameliorates posttraumatic excessive daytime sleepiness.		Kaiser et al., 2010
**Methylphenidate**	Increases the release of dopamine and inhibits its reuptake.	Cognitive disorders: improves attention, mental fatigue, working memory, executive functions, and processing speed.	Headache, insomnia, hyporexia, nausea, anxious feelings, increased heart rate and blood pressure.	Johansson et al., 2015, 2017; Manktelow et al., 2017; Zhang and Wang, 2017; Dorer et al., 2018; Espadas et al., 2018; Chien et al., 2019; Al-Adawi et al., 2020; Barnett and Reid, 2020
**Bromocriptine**	D_2_R agonist.	Stimulates alertness in patients with altered states of consciousness such as unresponsive wakefulness syndrome or a minimally conscious state.	Hypotension, nausea, vomiting, confusion, constipation, dizziness.	Celik et al., 2015; Munakomi et al., 2017; Otero Villaverde et al., 2019
		Ameliorates cognitive disturbances.		Munakomi et al., 2017
		Ameliorates central hyperthermia.		Zakhary and Sabry, 2017
**Apomorphine**	Non-selective D_2_R and D_1_R receptor agonist.	Stimulates alertness in patients with altered states of consciousness such as unresponsive wakefulness syndrome or a minimally conscious state.	Nausea, vomiting, headache, abnormal movements, hallucinations.	Fridman et al., 2010; Auffret et al., 2019
**Rotigotine**	Non-selective D_1_R to D_5_R agonist with the highest affinity for D_3_R.	Stimulates alertness in patients with altered states of consciousness such as unresponsive wakefulness syndrome or a minimally conscious state.	Changes in blood pressure, drowsiness, hallucinations, fainting, fluid retention.	Lan et al., 2019; Corsi et al., 2020
**Pergolide**	Non-selective D_2_R and D_1_R agonist.	Cognitive disturbances: improves working memory.	Dyskinesia, hallucinations, disturbance of sleep, loss of appetite, nausea, hypotension, tachycardia.	Flashman et al., 2021
**Levodopa**	Dopamine precursor.	Stimulates alertness in patients with altered states of consciousness such as unresponsive wakefulness syndrome or a minimally conscious state. Used as an adjuvant therapy for motor recovery.	Dyskinesia, nausea, vomiting, hypotension, mania, hallucinations.	Matsuda et al., 2005; Ennis et al., 2013; Bradley and Damiano, 2019

### Functional Interactions Between DA and Other Neurotransmission Systems in the Striatum

**FIGURE 3:**
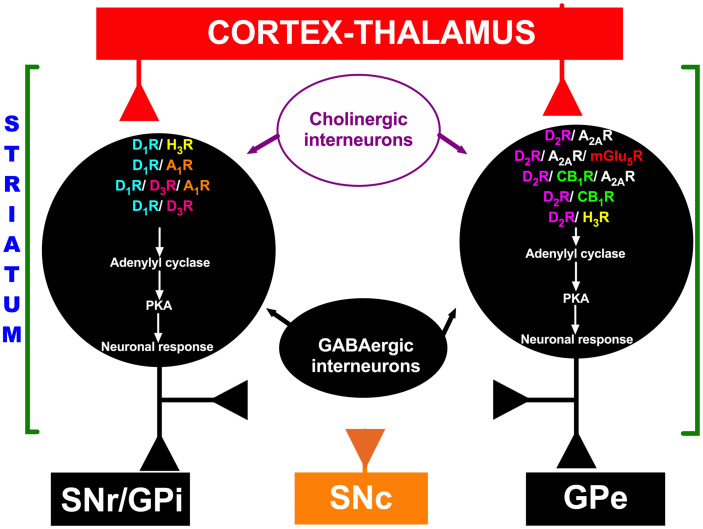
Schematic representation of functional interactions between DA receptors and other heteroreceptors localized in medium spiny neurons (MSNs). Red indicates excitatory projections, and black indicates inhibitory projections. In the striatum, GABAergic MSNs communicate with neurons in the substantia nigra pars reticulata (SNr) or internal segment of the globus pallidus (GPi) through a direct pathway and to the external segment of the globus pallidus (GPe); the GPe in turn projects to the subthalamic nucleus (STN), which projects to the SNr, forming the indirect pathway. The orange color represents the substantia nigra pars compacta (SNc). D_1_R, D_1_ receptor; D_2_R, D_2_ receptor; A_1_R, A_1_ receptor; A_2_R, A_2_ receptor; H_3_R, H_3_ receptor; D_3_R, D_3_ receptor; mGlu_5_R, mGlu_5_ receptor; CB_1_R, CB_1_ receptor; PKA, protein kinase A.

In the striatum, MSN heteroreceptors are localized in the cell membrane, including DA receptors (D_1_Rs and D_2_Rs), histamine receptors (H_3_Rs), muscarinic receptors (M1 and M4), adenosine receptors (A_1_Rs and A_2__*A*_Rs), cannabinoid receptors (CB_1_Rs), metabotropic glutamate receptors (mGlu_1__/__5_Rs), and ionotropic glutamate receptors [*N*-methyl-D-aspartic acid (NMDA) receptors, NMDARs] (Jones et al., 2001; Meschler and Howlett, 2001; Kreitzer and Malenka, 2008; Bolam and Ellender, 2016; Rapanelli, 2017). As mentioned above with regard to the role of DA after TBI and during functional recovery, studies have shown that functional interactions exist between DA receptors and other heteroreceptors localized in MSNs of the striatum, such as D_1_R/A_1_R (Fuxe et al., 2008, 2010), D_1_R/D_3_R/A_1_R (Fuxe et al., 2008), D_1_R/D_3_R (Fuxe et al., 2008; Marcellino et al., 2008), D_1_R/H_3_R (Garcia et al., 1997; Ferrada et al., 2009; Moreno et al., 2011; Avila-Luna et al., 2019), D_2_R/A_2__*A*_R (Fuxe et al., 2008, 2010; Ferré et al., 2016; Ferré and Ciruela, 2019), D_2_R/H_3_R (Humbert-Claude et al., 2007; Ferrada et al., 2008; Rapanelli et al., 2016; Rapanelli, 2017), D_2_R/A_2__*A*_R/mGlu5R (Fuxe et al., 2008, 2010), D_2_R/NMDA (Fuxe et al., 2008), D_2_R/CB_1_R (Fuxe et al., 2008), and D_2_R/CB_1_R/A_2__*A*_R (Meschler and Howlett, 2001; Fuxe et al., 2008, 2010) interactions (see Figure 3). Most of these functional interactions regulate neuronal activity via the adenylyl cyclase-mediated response in MSNs from both direct and indirect pathways of the basal ganglia (see Figure 3), but other interactions, such as D_2_R/NMDAR interactions, are associated with modulation of transmembrane ion currents (Fuxe et al., 2008). One explanation for the relevance of functional interactions between receptors to TBI-mediated effects involves the D_1_R/H_3_R functional interaction in MSNs, as it has been reported that H_3_R activation by the agonist immepip selectively inhibits the component of depolarization-evoked GABA release that depends on concomitant D_1_R stimulation in rat striatum and substantia nigra pars reticulata (SNr) slices (Garcia et al., 1997; Arias-Montaño et al., 2001). At the postsynaptic level, H_3_R activation inhibits D_1_R-induced cAMP formation in rat striatal slices (Sanchez-Lemus and Arias-Montano, 2004), and D_1_Rs play a permissive role in H_3_R-mediated activation of mitogen-activated protein kinases (MAPKs) (Moreno et al., 2011). At the motor behavior level, chronic H_3_R activation reduces dyskinesias induced by L-Dopa (L-3,4-dihydroxyphenylalanine) in rats with 6-hydroxydopamine-induced lesions (Avila-Luna et al., 2019). All of the functional interactions mentioned above may have important implications for future research on TBI and its treatment.

## Clinical Functional Recovery Associated With the Dopaminergic System

By ∼10 or ∼20 years after injury, most patients with moderate and severe TBI show good recovery or moderate disability (Andelic et al., 2018). Facilitation or modulation of functional recovery is an important goal of rehabilitation in all patients with severe TBI, but the degree of recovery varies among patients due to factors associated with the injury and to the patients’ biological and physical characteristics. One common limitation in the published studies is a small sample size. In this context, we considered illustrative examples from studies included in this review that provide evidence for an association between functional recovery and the dopaminergic system.

Pharmacological and physical rehabilitation is commonly utilized to treat patients with TBI (Horn et al., 2015; Bhatnagar et al., 2016; Ng and Lee, 2019; Traeger et al., 2020). As mentioned previously, dopaminergic drugs are often administered after brain injury. The evidence for an association of the dopaminergic system with functional recovery is described below. In the dopaminergic system, the nigrostriatal pathway is implicated in spatial learning/memory, reward processing and cognitive function, whereas the mesocorticolimbic pathway is associated with memory consolidation, motivation and addiction (Osier and Dixon, 2016a). A review by Osier and Dixon has suggested that therapies targeting the catecholaminergic system may attenuate functional limitations after moderate and severe TBI (Osier and Dixon, 2016a) and functional disability that may persist even decades after brain injury (Andelic et al., 2018).

Evidence indicates that DA neurotransmission is disrupted following moderate and severe TBI, and benefits associated with dopaminergic system-affecting drugs have been reported (Passler and Riggs, 2001; Meythaler et al., 2002; Sawyer et al., 2008; Bales et al., 2009; Jenkins et al., 2016, 2018; Osier and Dixon, 2016a; Lan et al., 2019). For example, administration of amantadine at 100–200 mg (twice a day) to patients with TBI appears to be beneficial in promoting intermediate-term cognitive recovery (Loggini et al., 2020). Administration of methamphetamine, which is highly addictive and is of limited usefulness due to its potential for abuse and dependence, to patients with TBI improves Glasgow Coma Scale (GCS) and Glasgow Outcome Scale (GOS) scores, whereas in injured rats, it improves motor and cognitive performance in a dose-dependent manner (Duong et al., 2018). Bromocriptine enhances arousal in patients with TBI who are in a minimally conscious state and improves neurological sequelae associated with hemiparesis (56% of cases), aphasia (80%), memory (50%), and cognitive impairment (67%) (Munakomi et al., 2017). Administration of MPD at a dose of 0.3 mg/kg (∼2.5 mg) exerts positive effects on attention deficits after TBI (Whyte et al., 2004).

### Clinical Evidence Associated With the Dopaminergic System

Clinically, a variety of drugs with dopaminergic effects are used for TBI. These drugs have diverse dosing and application regimens and clinical indications. A prudent approach would be to first describe two main categories of drugs that differ on the basis of their clinical use. The first category includes drugs that are used as adjuvant stimulant treatments during the rehabilitation or recovery period in patients with TBI. The expected (at least theoretically) effects of these drugs in modulating neuroplasticity processes are cumulative and long-term; such effects result in improved functional recovery of patients during follow-up (Ghalaenovi et al., 2018). The second category includes drugs that are administered to treat any symptoms or complications that patients are currently experiencing, for example, behavioral disturbances (aggressiveness and irritability), mood or motivational disorders (depression, apathy, etc.) and cognitive complaints (attention, posttraumatic amnesia, etc.) (Ter Mors et al., 2019).

The drugs that have been most commonly used in the clinic include amantadine, levodopa, amphetamine, MPD, bromocriptine and pergolide, among others. These drugs differ widely in their pharmacokinetic properties, mechanisms of action and selectivity for the dopaminergic system (Table 1; Lan et al., 2019). On the other hand, drugs with dopaminergic action have been used to treat various clinical conditions; for example, stimulants have been used in patients with disorders of consciousness, including patients with unresponsive wakefulness syndrome and patients in a minimally conscious state (Gao et al., 2020); for the treatment of some cognitive complaints such as attention disorders, posttraumatic amnesia, and executive or working memory dysfunction (Whyte et al., 2008); for management of behavior or mood alterations, such as aggressiveness, apathy, and agitation (Hammond et al., 2017); for stimulation of motor recovery; and even for management of autonomic dysfunctions secondary to TBI (Ghalaenovi et al., 2018). These drugs have mainly been administered to patients with severe TBI sequelae; however, reports have also described their use in patients with mild TBI (Iaccarino et al., 2020). The evolution of the injury is also highly variable, namely, the evolution from the acute or subacute stage to the chronic stage, which undoubtedly influences the clinical results obtained (Hammond et al., 2017). Additionally, the duration of treatment and the doses used have varied widely among different studies. For some drugs, such as amantadine, systematic reviews or meta-analyses have been published because numerous studies are available (Loggini et al., 2020); in contrast, for other drugs, only anecdotal reports of their use in case reports or case series are available. Finally, although multiple studies have shown positive effects of dopaminergic drugs, studies have also reported negative results; thus, the evidence is not entirely consistent in this regard (Hammond et al., 2017). A detailed description of each of the dopaminergic drugs and their clinical effects on TBI is beyond the scope of this review, but some recent publications on this topic are available (Carrillo-Mora et al., 2017; Bradley and Damiano, 2019). This variability among studies increases the difficulty of reaching categorical conclusions on the usefulness of dopaminergic drugs for TBI treatment in the clinical setting; however, the currently available evidence appears to suggest that they improve some disorders in patients with TBI, such as attention and alertness disorders, aggressiveness, and other behavioral symptoms. However, more clinical studies are needed to provide stronger support for their positive effects.

It is very important to emphasize that in the clinical management of TBI patients, in addition to using dopamine agonists for different purposes, clinicians frequently use dopamine antagonists (antipsychotics; either typical or atypical, which have different selectivities for D_1_Rs and D_2_Rs) to control some acute symptoms/complications, such as agitation, delirium, hallucinations and psychosis (Polich et al., 2019; Williamson et al., 2019). However, also importantly, there is experimental and clinical evidence that some of these dopaminergic blockers may have negative effects on neuroplasticity processes, affecting motor or cognitive recovery in patients with brain damage (Phelps et al., 2015; Folweiler et al., 2017). In this sense, previous studies have suggested that atypical antipsychotics have better safety profiles than typical antipsychotics (Phelps et al., 2017). Interestingly, this evidence regarding the negative effects of dopamine antagonists on motor or cognitive recovery from brain damage also indirectly supports the hypothesis that dopamine stimulation is a good therapeutic strategy for stimulation of motor and cognitive recovery after TBI; however, more studies are needed to confirm or rule out these negative effects of antipsychotic drugs.

## Conclusion

Facilitation or modulation of functional recovery is an important goal of rehabilitation in all patients after severe TBI, but the degree of recovery varies among patients due to factors associated with the biological and physical characteristics of the patients, the variety of the drugs and of the dosing and application regimens used, and clinical indications. A limitation of most studies is a small sample size. In this context, we considered illustrative examples from studies that were included in this review and provided some evidence for functional recovery associated with the dopaminergic system. Importantly, the dopaminergic nigrostriatal pathway is implicated in spatial learning/memory, reward processing, cognitive function and motor function, whereas the mesocorticolimbic pathway is associated with memory consolidation, motivation and addiction. In this review, we have described experimental and clinical evidence indicating that DA neurotransmission is disrupted following TBI and discussed the benefits associated with administration of dopaminergic system-affecting drugs. Ultimately, our review shows that TBI-induced disability may be partially associated with alterations in nigrostriatal dopaminergic signaling in the striatum that are localized in the basal ganglia and establish reciprocal interconnections with the corticostriatal pathway.

## Author Contributions

AB-N, AV-M, PC-M, and AA-L: drafting and refining of the manuscript. DM-R, AG-R, and AO-H: critical reading of the manuscript. All authors: read and approved the manuscript.

## Conflict of Interest

The authors declare that the research was conducted in the absence of any commercial or financial relationships that could be construed as a potential conflict of interest.
